# The automated processing algorithm to correct the test result of serum neuron‐specific enolase affected by specimen hemolysis

**DOI:** 10.1002/jcla.23895

**Published:** 2021-07-07

**Authors:** Xiao‐min Liu, Xiao‐hua Liu, Min‐jie Mao, Yi‐jun Liu, Jun‐ye Wang, Shu‐qin Dai

**Affiliations:** ^1^ State Key Laboratory of Oncology in South China; Collaborative Innovation Center for Cancer Medicine Guangzhou China; ^2^ Screening Department of Cancer Prevention Sun Yat‐sen University Cancer Center Guangzhou China; ^3^ Department of Medicine Laboratory Sun Yat‐sen University Cancer Center Guangzhou China; ^4^ Thoracic Surgery Department Sun Yat‐sen University Cancer Center Guangzhou China

**Keywords:** correction, equation, hemolysis, neuron‐specific enolase, serum

## Abstract

**Introduction:**

Serum neuron‐specific enolase (NSE) is an important tumor marker for small cell lung cancer and neuroblastoma. However, the test of serum NSE compromised by specimen hemolysis is presented as a falsely higher result, which seriously disturbs clinical decision. This study aimed to establish a solution integrated with laboratory information system to clear the bias from hemolysis on serum NSE test.

**Methods:**

The reference range of serum hemolysis index (HI) was first established, and specimen hemolysis rate was compared between HI test and visual observation. NSE concentration in serum pool with normal HI was spiked with serial diluted lysates from red blood cells to deduce individual corrective equation. The agreement between individual corrective equation and original NSE test was assayed by Bland and Altman plots.

**Results:**

The high HI existed in 32.6% of specimens from patients. The NSE median of hemolyzed specimens was significant higher than the baseline (*p* = 0.038), while the corrected NSE median had no difference compared with the baseline (*p* = 0.757). The mean difference of corrected NSE and initial NSE was 1.92%, the SD of difference was 5.23%, and furthermore, the difference was independent of tendency of HI (Spearman *r* = −0.069, *p* = 0.640). The 95% confidence interval of mean difference (from −8.33% to 12.17%) was less than the acceptable bias range (±20%).

**Conclusion:**

The agreement between individual correction equation and NSE assay was satisfied. Our automated processing algorithm for serum NSE could provide efficient management of posttest data and correct positive bias from specimen hemolysis.

## Graphical abstract

The automated processing algorithm for serum NSE influenced by specimen hemolysis. We recommend estimation for clinical needs when a NSE result is above 16.3 μg/L and is also influenced by specimen hemolysis. If the result is used for tendency supervision of tumor marker, level variation more than 25% compared to last test, which is warning clinical importance, will reminder the need of the individual corrected equation. And so it is if the result is used for auxiliary diagnosis of SCLC or NB.

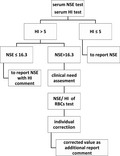

## 1 INTRODUCTION

Serum neuron‐specific enolase (NSE) presented exclusively in neurons and neuroendocrine tissues^1^, ^2^, ^3^ is an important tumor marker aiding to diagnose cancer of the neuroendocrine type, in particular small cell lung cancer (SCLC)^4^ and neuroblastoma (NB).^5^, ^6^ Subsequent to diagnosis, NSE is more frequently applied to evaluate the effect of therapy and to monitor metastasis or relapse of SCLC and NB.^7^ In addition, serum NSE plays a paramount role in the differential diagnosis of causes of dementia,^8^, ^9^ for assessment of the severity of traumatic brain injuries,^10^, ^11^ and for prognostication of likelihood of recovery after hypoxic ischemic brain injury.^12^, ^13^ It is very important for clinical laboratory to provide a precise NSE measurement result. However, the value of serum NSE measurement is easily influenced by specimen hemolysis due to more abundant NSE in circulating red blood cells (RBCs) and platelets.^3^, ^14^ The hemolysis brings a higher false result, which disturbs clinical decision. It is well known that specimen hemolysis dominated the majority of pre‐analytic error (from 40% to 70%) in clinical laboratories.^15^, ^16^ In face of hemolysis influence on familiar biochemical analytes, such as potassium, some clinical laboratories prefer to reject the result and recollect specimen,^17^ whereas some support to release the result with or without evaluation of the degree of hemolysis.^18^ As for NSE, it is more urgent to resolve this dilemma, and because serum NSE magnitude is thousand times less than that of potassium, specimen hemolysis brought striking great influences on serum NSE. It is reported the positive interference would occur when cell‐free hemoglobin concentration in serum is above 0.338 g/L at which visual inspection failed to identify.^4^

Clinical researchers are looking forward to establish an effective corrective formula in order to minimize hemolysis influence from specimen. First of all, the degree of hemolysis should be tested. The measurement method of micro‐cell‐free hemoglobin in serum has been developed from visual inspection,^19^ manual spectrophotometer^20^ to automatic biochemistry analyzer whose results can be processed by the laboratory information system (LIS).^21^ Visual inspection is an unreliable approach for assessing sample hemolysis, since it is arbitrary, not traceable and also plagued by poor sensitivity.^22^ Recently, cell‐free hemoglobin in serum is automatically quantified by absorbance measurements at different wavelengths on laboratory biochemistry analyzer, and the concentration of cell‐free hemoglobin is finally reported as “hemolysis index” (HI).^23^ HI values have a nearly linear correlation with increasing amounts of hemolysis as defined by free hemoglobin concentrations. Furthermore, it was ascertained that the ratio of NSE to HI in RBCs was statistically different among individuals.^20^ In this study, we aimed to establish an individual formula incorporating HI and the ratio of NSE to HI in RBCs to clear hemolysis influence on serum NSE test and define the possibility of applying on clinical practice.

## 2 MATERIAL AND METHODS

### 2.1 Study objective and specimens

All specimens involved in this study were detected by routine blood examination and tumor marker examination ordered by doctors. Informed consent about research use of left‐over routine samples was signed by every inpatient, and this study was approved by Institutional Review Board and Human Ethics Committee of Sun Yat‐Sen University Cancer Center.

To establish the reference range of hemolysis index (HI), serum control came from 200 healthy individuals that attended annual checkup at the screening department of cancer prevention. Serum pools were needed to deduce the corrective equation, and samples from 503 inpatients with NSE concentrations distributing from below 16.3 μg/L–370 μg/L were collected in 3 months. These serum samples were also used to compare the sensitivity for hemolysis recognition between visual observation and HI test. The performance of individual corrective equation was evaluated on 47 specimens from patients including 9 SCLC, 10 NSCLC, 7 NB, 16 other types of cancer, and 5 benign lung diseases. EDTA‐K2 whole blood specimen must be handled within 24 h to obtain concentrated lysate of RBCs. All serum samples and concentrated RBCs lysates were stored in −80°C.

### 2.2 Measurement of serum NSE

The serum NSE was tested by electrochemiluminescence immunoassay on Cobas e 602 analyzer (Roche Diagnostics, Mannheim, Germany). According to the manual, the 95^th^ percentile of reference range is 16.3μg/L, and the measurable range is from 0.05μg/L–370μg/L.

### 2.3 Measurement of serum hemolysis index (HI)

Control specimen was drawn into inert separation gel vacuum collective tube by expert nurse according to the standard collection procedure. One specified laboratory technician got carefully the specimen back to the screening department laboratory, avoiding shake and jolt. The specimen was centrifuged at 1,300 ×*g* for 10 min at room temperature within 1 h from collection (according to the manual) and then was analyzed for serum NSE within 2 h from collection.

The HI was analyzed on Cobas c 702 biochemistry analyzer (Roche Diagnostics, Mannheim, Germany) by paired bichromatic wavelengths using Serum Index Gen2 kit (Roche Diagnostics, Mannheim, Germany). It was calculated as the difference of absorbance at 570 nm and 600 nm converted into absolute number (range: 1–1,000). The semi‐quantitative value of HI corresponded to free serum hemoglobin (Hb) concentration (1 HI =10 mg/L Hb).^21^ The repeatable precision and intermediate precision were 2% and 5%, respectively.

### 2.4 Sensitivity comparison of hemolysis recognition between visual observation and HI test

For serum sample with HI above 95^th^ percentile of reference range, the hemolysis was independently classified by two technicians as the following four degrees: no hemolysis, slight hemolysis, hemolysis, and severe hemolysis. The HI test and hemolysis degree were finished in 1 day for all specimens. In addition, the severe hemolyzed serum determined by visual observation among all specimens in the laboratory was further analyzed for peak HI.

### 2.5 Analyzation of NSE/Hb ratio

Concentrated RBCs lysate was prepared according to the published study.^20^ It was then diluted by normal saline according to the ratio of 1:30 and centrifuged at 1,800 ×*g* for 10 min to remove cellular debris. The supernatant served as diluted RBCs lysate ready for measurement of NSE and HI to obtain NSE/HI ratio.

### 2.6 Derivation of individual corrective equation for NSE in hemolyzed serum

Specimens with normal HI out of 503 patients were chosen from 23 serum pools whose baseline NSE concentrations ranged from low to high. Each serum pool was divided into 11 aliquots. Concentrated RBCs lysate from one healthy individual was used as spiking NSE origination, which was diluted with normal saline to serial HI concentrations: 6,000, 3,000, 1,500, 750, 375, 187.5, 93.75, 46.87, 23.43, and 11.71. These 10 serial diluted RBCs lysates were added into 10 aliquots of one serum pool according to 1:10 ratio. Normal saline was added into the eleventh aliquot serum, serving as baseline serum. 20 out of 23 serum pools were spiked according to the method. To improve the accuracy of corrective performance for baseline serum NSE concentration below 16.3μg/L, 3 serum pools were spiked to serial HI concentrations: 5,000, 2,500, 1,250, 625, 312.5, 156.25, 78.13, 39.06, 19.53, and 9.76.

The resulting 253 (23 × 11) samples were measured for HI and NSE within 1 day. Actual increased NSE and HI originating from hemolysis were obtained by subtracting NSE and HI in baseline serum from measurement NSE (NSE_meas_) and measurement HI (HI_meas_), respectively. Corrective equation was achieved by nonlinear fit.

### 2.7 Performance validation of individual corrective equation

We evaluated the performance of individual corrective equation on specimens from 47 inpatients. Serum of patient's specimen that had finished NSE examination served for baseline self‐control. Then the specimen was intentionally hemolyzed by the following procedures: collect some (30 μl–100 μl) RBCs beneath separation gel, mix the RBCs with serum above separation gel, then aspirate 100 μl mixture of serum and RBCs to proceed two cycles of refrigeration, thaw and vibration to break the RBCs, and transfer the supernatant into the specimen. After centrifugation, the intentionally hemolyzed specimen was finished for testing serum NSE and HI.

The NSE/Hb ratio of RBCs from these inpatients was also analyzed individually. The NSE concentration values from baseline serum, intentionally hemolyzed serum, and individual correction were compared. The agreement between baseline NSE concentration and individual corrected concentration by the equation was evaluated by Bland and Altman method. The 95% limit of agreement for bias was considered acceptable if it fell within ±20%. The cutoff was chosen because the intermediate precision of NSE assay was CV ≤10.0% according to the reagent manual, where meant approximately 95% of repeat results would be expected to fall within ±20% of the original result. Furthermore, the difference within 20% of serum NSE concentration is not clinically important for supervising relapse or metastasis of tumor.

### 2.8 Statistical analysis

Statistical analysis was carried out by GraphPad Prism5.0. The distribution of data set was measured by KS normality test. The serum HI and NSE concentration were described by median and interquartile range, and Mann‐Whitney test was used to compare between groups for serum HI or serum NSE concentrations. Reference range was defined according to the Clinical and Laboratory Standards Institute (CLSI) document C28‐A3.^24^ Nonlinear regression was used to ascertain the relationship of serum HI and NSE concentration increment from RBCs. The Bland and Altman plots were used to evaluate the agreement of two measurement methods. Statistically significant was considered when a *p* < 0.05 for all statistical comparisons.

## 3 RESULTS

### 3.1 Establishment of the reference range of serum NSE‐specific HI

Serum NSE from the 200 cases of healthy control individuals were all below 16.3 μg/L, the median was 8.5 μg/L, and the 95^th^ percentile was 11.8 μg/L which was lower than 16.3 μg/L defined in the manual. The HI reference range (95%) was from 0 to 5, which was in accordance with HI operating manual, and the median of serum HI specific to NSE assay was 2.

### 3.2 Abnormal HI presented in 32.6% of inpatients' serum

**FIGURE 1:**
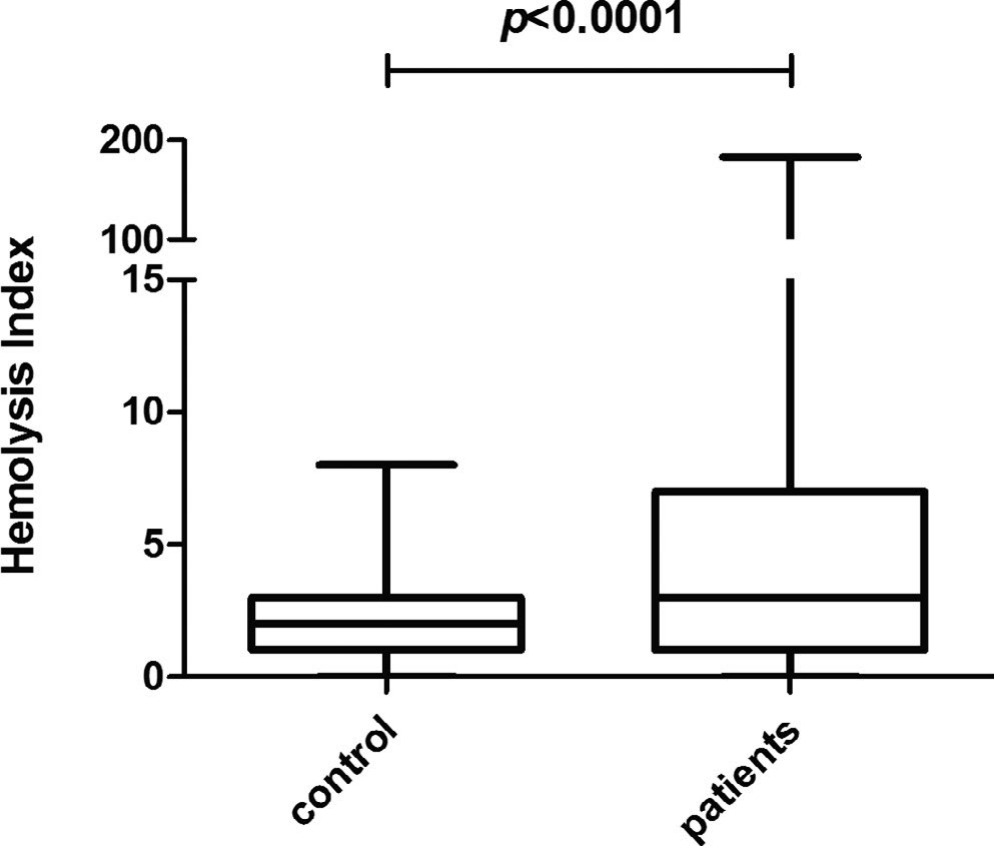
Hemolysis index value of serum specimens from patients was significantly higher than that from controls by Mann‐Whitney test

The serum HI values of inpatients were significantly higher than that in healthy control (*p* < 0.0001, Figure 1). According to HI reference range we have established, abnormal HI was defined as above 5. Generally, abnormal HI (32.6%, 164/503) may occur in serum with various levels of NSE concentrations (Table 1), and the median of abnormal HI value was 11 (equivalent to 110 mg/L free hemoglobin) which was far less than the visual detectable hemolysis limit of 300 mg/L free hemoglobin. The abnormal serum HI rate was 21.7% (47/217) even if NSE was below 16.3μg/L. As for NSE results between 16.3 μg/L and 50.0 μg/L which brought most confusion to clinicians, the abnormal HI rate reached amazingly 45.0% (95/211), meanwhile the maximum HI value of 183 occurred in this group.

**TABLE 1:** Descriptive statistics of abnormal hemolysis index of 503 samples from inpatients with different serum NSE levels (μg/L)

	NSE ≤16.3	16.3 <NSE ≤50	50 <NSE ≤100	100 <NSE ≤370
Sample size	217	211	43	32
Samples hemolyzed	47 (21.7%)	95 (45.0%)	11 (25.6%)	11 (34.4%)
Hemolysis index				
Minimum	6	6	6	7
25% percentile	6	9	7	10
Median	7	15	14	13
75% percentile	10	24	33	15
Maximum	48	183	82	18

Abbreviation: NSE, neuron‐specific enolase.

We chose 147 samples with HI >5 to be inspected visually for the hemolysis degree by two technicians independently. Although the consistency between technicians was satisfactory (*p* = 0.38), HI was far more sensitive than visual observation (*p* < 0.0001) by Fisher's exact test, and 76.8% (113/147) of serum with abnormal HI could not be identified by visual observation.

### 3.3 Corrective equation for hemolyzed serum NSE concentration must introduce personal variable: NSE/HI ratio

**FIGURE 2:**
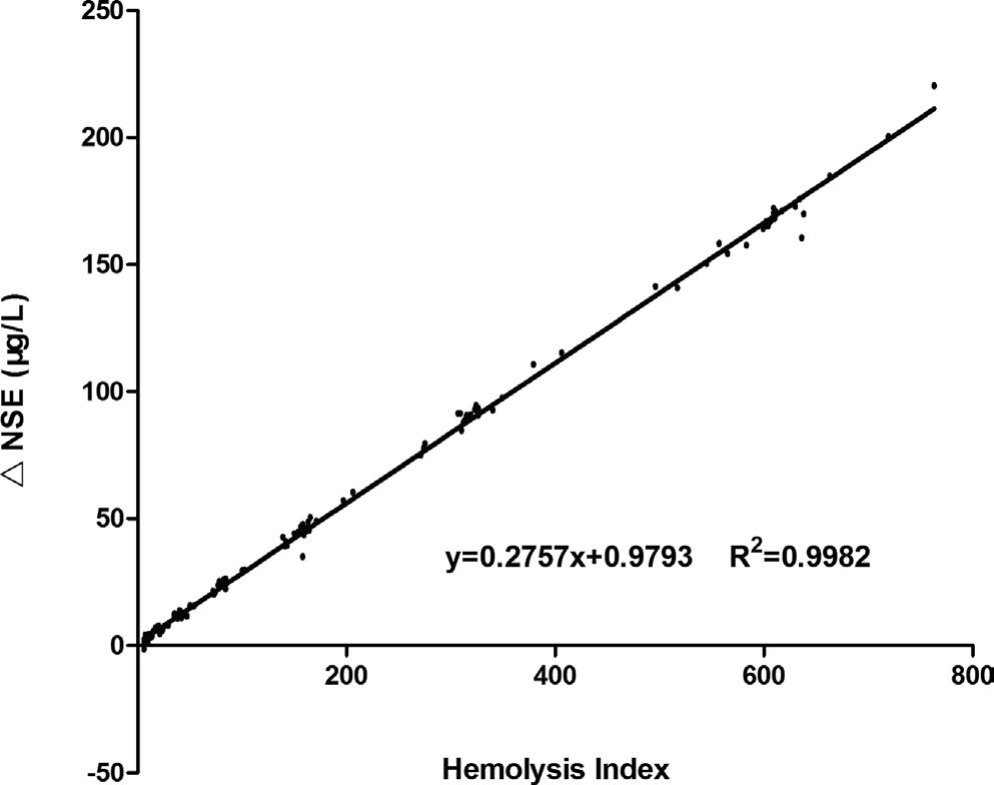
Actual NSE concentration increment (μg/L) was positive correlated with the HI value in serum sample

After spiking with RBCs lysate, 165 samples with HI >5 out of 230 serum pools contributed data pairs of NSE and HI to deduce formula. By one step nonlinear fit, a straight line equation (Figure 2) was determined for actual NSE concentration increment (*y*) plotted as a function of *HI_meas_ (x)*:

y=0.2757×HImeas+0.9793

This corresponds to 4.0 μg/L false elevated NSE originating from RBCs lysate when serum HI was 11. But it was a pity that NSE/HI ratio (*R*) was not introduced into this equation. The increased NSE amounts from hemolysis were theoretically different when two individuals with different NSE/HI ratios presented same serum HI value, and the difference would become larger following the increasing *HI_mea_*. Personal NSE/HI ratio (*R*) was already demonstrated more accurately than a mean of *R* from healthy individuals which was used as generalized R by some researches.^21^, ^25^ Hence a rational equation must introduce personal R as variable. When the slope and the intercept with y‐axis were introduced personal R (the R of RBCs in spiking test was 0.31), the above equation is shown as:

y=0.889×R×HImeas+3.159×R

Then, by introducing individual *R*, *HI_meas_* and *NSE_meas_* as variables, we obtained an individualized corrective equation for serum NSE influenced by hemolysis:

NSEcorr=NSEmeas‐y=NSEmeas‐0.889×R×HImeas‐3.159×R

### 3.4 The performance of individual corrective equation accorded with quality requirements

**FIGURE 3:**
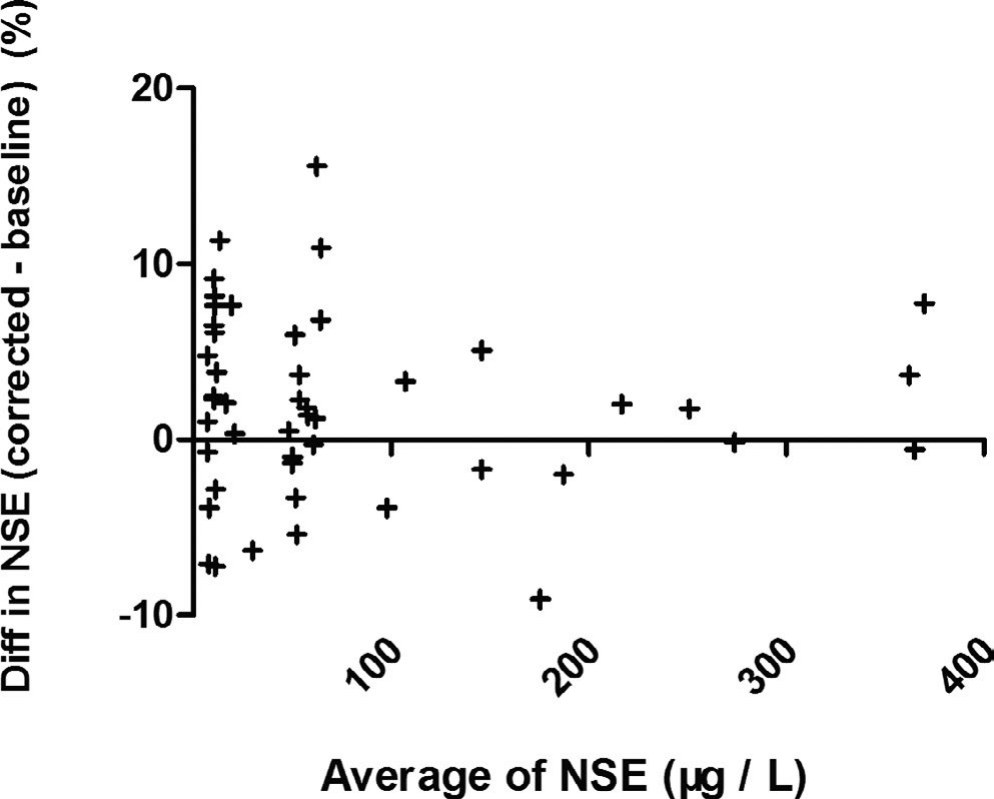
Bland‐Altman plots with the difference between corrected NSE and baseline NSE as a function of the average

We evaluated the equation performance of 47 specimens from validated patient group (Table 2). The validated HI range (6–314) covered the common abnormal HI range (6–183). The validated NSE level covered the measurable range of NSE assay. The NSE median of specimens hemolyzed was significant higher than the baseline (*p* = 0.038), while the corrected NSE median had no statistical difference compared with the baseline (*p* = 0.757). The agreement between individual correction equation and NSE assay was evaluated by Bland‐Altman plots (Figure 3). The mean difference of corrected NSE and initial NSE was 1.92%, the SD of difference was 5.23%, and the difference was independent of tendency of HI (Spearman r = −0.069, *p* = 0.640). The 95% confidence interval of mean difference was from −8.33% to 12.17%, which was less than the acceptable range of original NSE result (±20%), although the difference was slightly larger at lower NSE level than at higher level. The key of clinical application for a tumor marker focused on the level variation trend, at which the increase or decrease less than 25% had no clinical importance. These results indicated that individual correction could be an alternative option for serum NSE concentration which was influenced by specimen hemolysis.

**TABLE 2:** Descriptive statistics of spiked serum pools and validation specimens

	Spiked serum pools		Validation specimens				
	NSE	HI	NSE (baseline)	NSE (hemolyzed)	NSE (corrected)	HI (hemolyzed)	NSE/HI in RBCs
Sample size	165	165	47	47	47	47	47
Minimum	8.420	6	6.94	9.72	6.89	6	0.091
25% percentile	27.15	21	10.32	22.66	10.95	15	0.267
Median	61.68	79	50.46	60.91^*^	50.90^**^	36	0.309
75% percentile	136.8	309	99.87	112.8	96.07	69	0.337
Maximum	387.3	763	366.1	400.6	384.3	314	0.467

Abbreviations: HI, hemolysis index; NSE, neuron‐specific enolase.

^*^NSE level, hemolyzed samples vs. baselines, *p* = 0.038.

^**^NSE level, corrected level vs. baselines, *p* = 0.7565.

## 4 DISCUSSION

Carraro first investigated the causes for specimen hemolysis in 2,000 and proved that hemolytic specimens were mostly due, up to 96% of the cases, to in vitro hemolysis. By visual observation, about 3.3% of all specimens sent to clinical laboratory appeared hemolysis hue.^26^ Lippi also demonstrated 5.6% for hemolysis rate by visual observation in 2009.^27^ However, visible inspection with poor sensitivity brings low hemolysis rate. Clinically significant false‐elevations of NSE are observed even with visually undetectable hemolysis.^25^ One survey employing HI test however showed an overall rate of 10.4% for hemolysis in primary healthcare centers and even a 31.1% rate in the emergency department.^28^ Our data demonstrated that the visual inspection could only find minority of specimens that were hemolysis judged by HI test. Based on the serum HI reference we established, we proved 32.6% for hemolysis rate. In contrast to visual scrutiny, HI is reasonably more sensitive and more precise to find out more hemolysis specimens, especially invisible slight hemolysis interfering clinical decision. Furthermore, our study first established the HI cutoff value specially for serum NSE, which is the critical foundation to judge specimen hemolysis precisely and to utilize the individual corrective formula.

**FIGURE 4:**
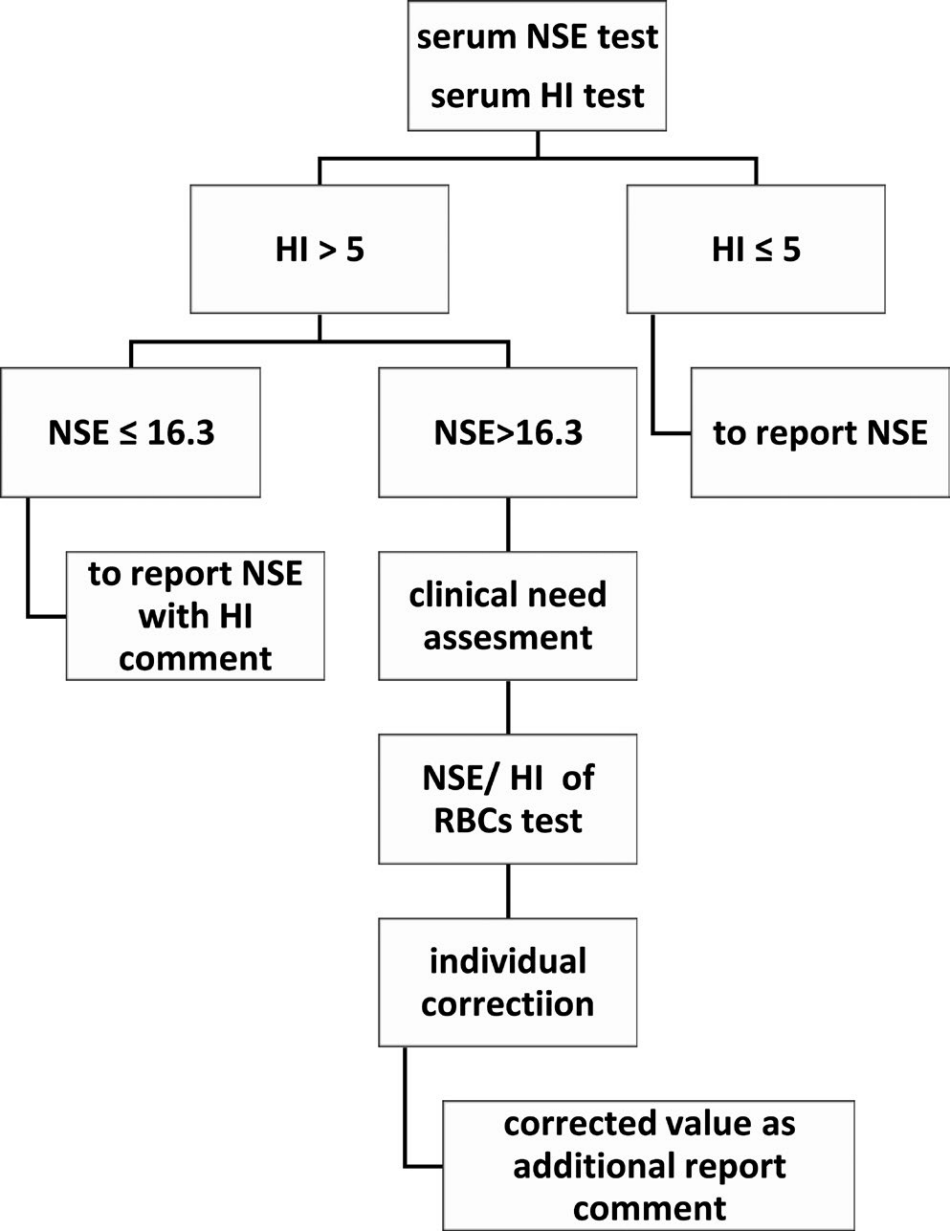
Automated processing algorithm for serum NSE influenced by specimen hemolysis

Now many clinical laboratories equip with continuous‐flow analytic system, yet the pre‐analytical module that can automatically centrifuge specimens and then transport specimens to analytical module, hides away all hemolysis specimens from naked eyes. The assessment of serum indices including HI has now become a mainstay for establishing sample quality,^29^ as also endorsed by the CLSI document C56‐A. The HI test which can be processed by LIS is very important for every specimen quality monitoring in continuous‐flow automation laboratory. In fact, many large hospitals have showed HI result on each biochemical report to clinicians. The agreement between NSE test and individual corrective equation by Bland‐Altman analysis was satisfied, and the HI range and NSE level for equation apply were also validated meeting clinical requirements. So the individual corrective equation could be used when the result of serum NSE was influenced by specimen hemolysis, in another word, the contribution of serum NSE individual correction of one hemolyzed specimen equals to retest another specimen redrawing. A corrected result could be offered to clinician for assessing original NSE level without hemolysis influence, avoiding time delay to recollect specimen and to retest. Hence, we recommend an automated processing algorithm for serum NSE influenced by specimen hemolysis (Figure 4). It is difficult for every hemolysis specimen to obtain a corrected result in a busy clinical laboratory due to the time‐consuming procedure of manually testing individual NSE/HI ratio of RBCs. Furthermore, alerting clinician only when a meaningful action should be taken could prevent “alert fatigue” by clinician. We recommend estimation for clinical needs when a NSE result is above 16.3 μg/L and is also influenced by specimen hemolysis. If the result is used for tendency supervision of tumor marker, level variation more than 25% compared to last test, which is warning clinical importance, will remind the need for the individual corrected equation. And so it is if the result is used for auxiliary diagnosis of SCLC or NB. The report may present clinician with the original level influenced by specimen hemolysis, also with the serum HI and the corrected level. In another study of HI, it was also supported that aligning report of sample hemolysis with clinically significant changes may provide clinically meaningful alerts regarding this common pre‐analytic error.^30^

The reference range of serum NSE remains controversial. The reagent manual from Roche company defines the expected value as lower than 16.3 μg/L (95th percentile) with a 95th confidence range from 15.7 μg/L–17.0 μg/L. However, the hemolysis was not mentioned about the study specimens in the manual. However, this reference range is usually questioned in clinical laboratory because many serum NSE results were unreasonably >16.3 μg/L. Qian Liu determined the reference upper limit of serum NSE as 25.4 μg/L (90% confidence interval: 24.5–26.2 ng/mL).^31^ Erfu Xie suggested the reference upper limit of serum NSE as 18.9 μg/L.^32^ The time intervals from blood collection to centrifugation were 2 h for research from Qian Liu and 3 h for research from Erfu Xie, respectively, although the time interval is within 1 h according to the manual. The longer the specimen is kept before centrifugation under room temperature, the more erythrocytes are broken. In our study, the specimens were centrifuged within 1 h from collection and were tested for HI, and our reference range is high in credibility by excluding specimen hemolysis.

## 5 CONCLUSION

In our research, the HI was tested by Roche Diagnostics automated. Other clinical laboratories probably tested HI on analytical platforms from different manufactures like Siemens, Beckman, etc. A multicenter evaluation study for HI test on different platforms demonstrated that overall imprecision of the instruments tested is satisfactory with inter‐assay CVs between 0.1% and 2.7%, and the reproducibility among different facilities using the same instrument (Roche, Modular System P) is excellent.^33^ Another research proved that automated HI quantification on Roche Modular clinical chemistry platform correlated well with results using the classical spectrophotometer methods, and recovery was good for self‐made controls.^34^ However, quantitative HI results provided by Roche and semi‐quantitative HI results provided by Beckman coexist, and more effort should be placed on the standardization and harmonization of HI reporting. Our automated processing algorithm for serum NSE influenced by specimen hemolysis may suit for laboratories with Roche continuous‐flow analytic system, while other laboratories need to develop their own ways to integrate HI information into LIS for personalized solution.

## CONFLICT OF INTEREST

The authors declare that they have no conflict of interest.

## AUTHOR CONTRIBUTIONS

Jun‐ye Wang and Shu‐qin Dai involved in conception and design. Shu‐qin Dai contributed to administrative support. Xiao‐min Liu and Xiao‐hua Liu involved in provision of study materials or patients; Min‐jie Mao contributed to collection and assembly of data. Yi‐jun Liu involved in data analysis and interpretation. All authors involved in manuscript writing and final approval of manuscript.

## Data Availability

The datasets analyzed during the current study are not publicly available due to patient privacy concerns, but are available from the corresponding author on reasonable request.
